# Eroding University Autonomy and Emerging Ethical Risks: Lessons From a Corruption Case Involving the University of Tokyo and the Japan Cosmetic Association

**DOI:** 10.34172/ijhpm.9561

**Published:** 2026-03-14

**Authors:** Akihiko Ozaki, Yudai Kaneda, Masahiro Kami

**Affiliations:** ^1^Breast and Thyroid Center, Jyoban Hospital of Tokiwa Foundation, Fukushima, Japan.; ^2^Clinical Training Center, Jyoban Hospital of Tokiwa Foundation, Fukushima, Japan.; ^3^Medical Governance Research Institute, Tokyo, Japan.

## Dear Editor,

 Universities must sustain intellectual and ethical independence, which depends in part on financial autonomy. Harvard University illustrates this model. As of 2025, its endowment totaled US $56.9 billion, generating annual distributions of US $2.5 billion within a total operating budget of US $6.8 billion.^1^ This financial structure has allowed Harvard to maintain institutional autonomy even under political pressure by limiting reliance on government funding.

 By contrast, Japanese national universities operate within a structurally dependent model. In 2021, the University of Tokyo received JPY 87 billion in government operating grants (US $785 million US, based on the 2021 annual average exchange rate of 110.8 JPY/USD), accounting for about 30% of its JPY 280 billion budget (around US $2.5 billion).^2^ At the same time, income from industry–academia collaboration and donations had grown to nearly JPY 69 billion (about US $623 million), while university hospital revenue reached approximately JPY 54 billion (around US $487 million).^2^ The scale and relative autonomy of hospital funding have historically limited central oversight, complicating institutional control over increasingly extensive industry relationships.

 Academia–industry collaboration can advance innovation when properly governed, but requires the ability to select and reject partners based on academic values—an ability that appears insufficient even at the University of Tokyo. In November 2025, the university was implicated in bribery cases in which donations from medical device companies were judicially recognized as kickbacks,^6^ and investigative reporting has shown that Philip Morris Japan covertly used academic collaborations to promote harm reduction narratives and heated tobacco products while concealing institutional ties.^7^ The university also leads MbSC2030 under the Saudi–Japan Vision 2030, despite ongoing international human rights concerns regarding Saudi Arabia.^8^

 As industry–academia collaboration continues to expand, scandals at the University of Tokyo offer critical lessons not only for Japanese higher education but for universities worldwide. This case study examines the January 2026 arrest of a senior professor of dermatology at the University of Tokyo on suspicion of bribery.

## Case

 According to court records and investigative reporting, the university entered into a joint research agreement with the Japan Cosmetic Association through a senior professor of dermatology, under which a cooperative research chair was established for the period from March 2023 to March 2026, with an annual research budget of ¥30 million (approximately US $212 000, based on the 2023 annual average exchange rate of 141.56 JPY/USD) to be borne by the association and an affiliated corporation.^9^ In April 2023, the chair was formally launched, with the professor serving as chair director and a specially appointed associate professor as chair head, while executives of the association were registered as research staff.^9^

 Between February 2023 and approximately mid-2024, the professor and the associate professor allegedly received frequent hospitality—reported to be two to four times per month—from the association’s representative director, including dining at high-end restaurants, clubs, hostess bars, and entertainment involving sexual services.^9^ Civil litigation filed by the association alleges that the professor alone received 86 instances of such hospitality, totaling approximately ¥20.8 million (around US $147 000), while the professor has acknowledged 31 instances amounting to roughly ¥9.2 million (about US $65 000) during investigative interviews.^9^

 In return, the professor allegedly provided preferential treatment to the association, including expanding the research scope beyond dermatological disease to encompass cannabinoid-based cosmetic product development.^9^ He also submitted multiple amendment applications that increased the total research budget from ¥90 million (US $636 000) to approximately ¥199 million (US $1.41 million) over three years.^9^ Although the contract stipulated that the association would fully fund the research, only ¥1 million (approximately US $7100) was ultimately transferred to the university.^9^

 As relations between the parties deteriorated in 2025, mutual accusations of misconduct led to criminal and civil proceedings. On January 24, 2026, the professor was arrested on suspicion of bribery; on January 26, the associate professor and the representative of the Japan Cosmetic Association were referred to prosecutors on suspicion of accepting and offering bribes, respectively, and on January 27, the director of the University of Tokyo Hospital resigned.

 At a press conference on January 28, the President of the University of Tokyo acknowledged that the unpaid research funds were identified only after a whistleblower report, stating that the university would not have noticed the issue otherwise.^9^ He cited three core failures: inadequate ethical awareness among faculty and staff, insufficient oversight of privately funded collaborative programs, and an insular, hierarchical organizational culture within the medical faculty and affiliated hospital that failed to detect or prevent misconduct.^9^

 The professor had long maintained substantial financial relationships with pharmaceutical companies; publicly available payment data show that between 2019 and 2023, he received approximately ¥51 million (about US $360 000) from pharmaceutical and medical device companies. These amounts placed him at the top of payment rankings among active professors at the university during that period, and while formally disclosed and legally permissible, they underscore how deeply normalized close financial ties between senior academics and industry had become.

 The Japan Cosmetic Association had already attracted controversy before its engagement with the university. Since around 2019, it positioned itself as an authority in the emerging cannabidiol (CBD) market by conducting independent product testing and publicly releasing results, in some cases without the consent of the companies involved, raising concerns about unaccountable quasi-regulatory influence.^10^ The association also operated a paid CBD certification system with substantial approval and renewal fees, and in the absence of comprehensive public regulation, affiliation with a prestigious academic institution may have served to legitimize its activities.^10^

## Discussion

 This case represents an exceptionally severe scandal in Japanese academic medicine, distinguished not only by the scale and nature of the misconduct but also by the University of Tokyo’s failure to intervene or exercise internal corrective action until the matter escalated into a criminal case.

 Remarkably, no effective internal corrective measures were taken against the professor prior to his arrest. Similarly, the university was unable to take disciplinary or corrective action internally even prior to the associate professor’s arrest in November 2025. This inability to detect and address misconduct before judicial involvement highlights a fundamental breakdown of internal governance at a time when universities are increasingly dependent on external funding and must therefore exercise heightened vigilance over industry relationships.

 This extraordinary scandal must be understood in the context of long-standing normalization of excessive financial relationships with pharmaceutical and medical device companies. The professor and even the hospital director who later resigned were among the highest recipients of industry payments. Such normalization likely eroded sensitivity to conflicts of interest and reduced resistance to progressively more problematic relationships, rendering the eventual misconduct neither sudden nor isolated.

 From a policy perspective, legal transparency mechanisms such as Open Payments databases may be useful, but this case arose through relationships with organizations outside the traditional pharmaceutical and medical device sectors, where legal frameworks are likely to be bypassed. Instead, responses should prioritize stricter limits on financial relationships involving academic physicians, particularly those in senior roles, together with strengthened internal governance through audits, compliance checks, and clearer accountability between universities and affiliated hospitals. More fundamentally, without a consistent ethical stance toward external funding and greater institutional autonomy, including reduced structural dependence on industry through increased education-related revenue such as tuition, organizational reforms alone are unlikely to prevent future misconduct.

## Disclosure of artificial intelligence (AI) use

 The authors utilized ChatGPT (GPT-5.2, OpenAI) to assist with English language ‎proofreading in the preparation of this manuscript‎.

## Ethical issues

 This study was based solely on publicly available open data and was exempt from review ‎by an institutional review board‎.

## Conflicts of interest

 Akihko Ozaki received personal fees from MNES, Kyowa Kirin Inc., Becton, Dickinson ‎and Company, Pfizer, Daiichi Sankyo Inc, and Taiho Pharmaceutical Co., Ltd., outside the ‎scope of the submitted work. Regarding non-financial conflicts of interest among the ‎study authors, Akihiko Ozaki is engaged in ongoing research examining financial and ‎non-financial conflicts of interest among healthcare professionals and pharmaceutical ‎companies in Japan‎.
